# Concomitant use of Sapien 3 transcatheter valve for severe MAC with Intuity Elite rapid deployment valve for aortic stenosis

**DOI:** 10.1186/s13019-022-01879-7

**Published:** 2022-05-23

**Authors:** Gareth J. Hooks, Peter Ball, Mark S. Spence, Reuben Jeganathan

**Affiliations:** 1 Department of Cardiothoracic Surgery, Royal Victoria Hospital, Belfast, BT12 6BA UK; 2 Department of Radiology, Royal Victoria Hospital, Belfast, UK; 3 Department of Cardiology, Royal Victoria Hospital, Belfast, UK

**Keywords:** TMVI, Concomitant rapid deployment aortic valve

## Abstract

**Background:**

Concomitant double valve pathology in the presence of severe MAC poses significant technical challenges when planning surgical intervention. With continued evolution of valve prosthesis, innovative techniques can be considered with the potential for additional therapeutic benefit.

**Case presentation:**

We present a novel technique of using a rapid deployment surgical aortic valve in combination with open surgical transcatheter mitral valve implantation (TMVI) for severe Mitral Annular Calcification (MAC). The Intuity Elite rapid deployment prosthesis (Edwards Lifesciences, Irvine, CA) was used concomitantly with the Sapien 3 (Edwards Lifesciences, Irvine, CA) transcatheter prosthesis trans-atrially on cardiopulmonary bypass in a patient with critical aortic stenosis and moderate-severe mixed mitral valve disease in the setting of severe MAC (off-label use).

**Conclusions:**

We demonstrate how both technologies can, not only be accommodated, but indeed complement each other achieving an excellent outcome in a high-risk patient.

## Background

With increasing experience in our unit of transcatheter mitral valve implantation (TMVI) for severe mitral annular calcification (MAC), it has become apparent that this high-risk patient cohort frequently present with additional valvular lesions. To develop a standardized approach, reduce cross-clamp duration and take advantage of the superior haemodynamic properties, we embarked on the concomitant use of an Intuity Rapid Deployment aortic valve.

## Case presentation

A 73-year-old gentleman was referred from the heart team meeting with known severe MAC for consideration of inpatient mitral and aortic valve replacement, NYHA III, CCS 0 rendering him housebound on a background of two hospital admissions within 6 weeks due to heart failure. Height 173 cm, weight 113 kg, body surface area 2.25m^2^. Cardiac risk factors include peripheral vascular disease, insulin dependent diabetes, elevated BMI (37.8 kg/m^2^), hypertension, atrial fibrillation and permanent pacemaker insertion for tachy-brady syndrome. Additional past medical history of significant asbestos exposure with wide spread pleural plaques, a large benign pleural based mass and asthma.

**Fig. 1:**
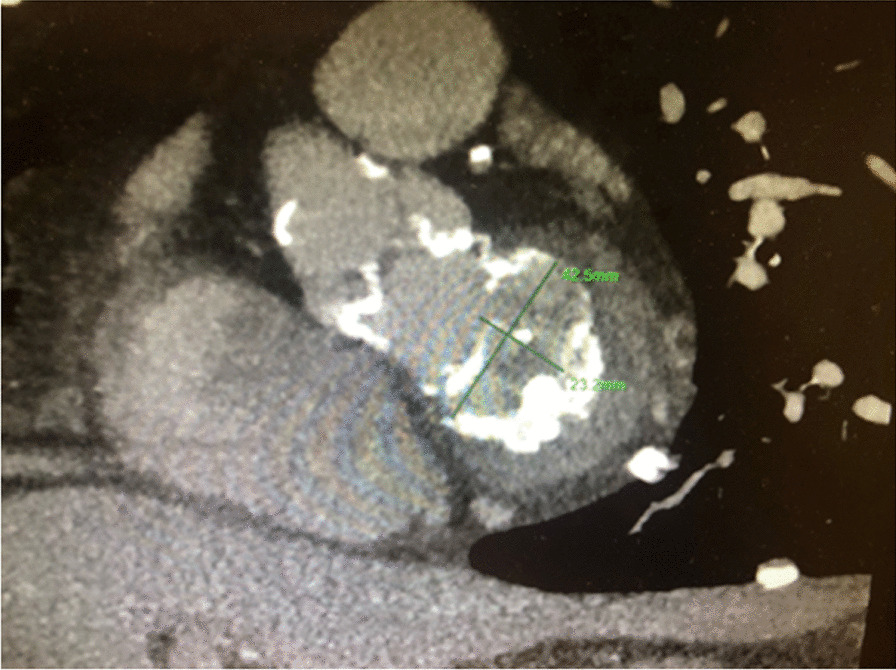
Cardiac-gated CT demonstrating severe mitral annular calcification with annular dimensions of 42.5 mm × 23.2 mm

Coronary angiography demonstrated a right dominant system with no obstructive disease and relatively high set coronary ostia (17 mm above the nadir of the coronary sinuses). Echocardiogram demonstrated mixed mitral valve disease with moderate-severe mitral stenosis (peak/mean gradient 20/9 mmHg) and moderate mitral regurgitation (MR), critical aortic stenosis (103/58 mmHg, AVA 0.6 cm^2^) with calcification extending down on to the aorto-mitral curtain and severe mitral annular calcification. Mild tricuspid regurgitation (TR) was also identified however the maximum annular dimension was 38 mm and surgical repair was not indicated. Preserved biventricular function was noted in the setting of MR and TR. A TAVI cardiac gated computed tomography demonstrated extensive MAC involving both trigones with extension onto the aortic-mitral curtain (Fig. 1). Mitral annular dimensions were 42.5 mm × 23.2 mm. A multi-disciplinary planning meeting with a consultant radiologist, consultant cardiologist and consultant cardiac surgeon was held to discuss surgical feasibility. As this was an open trans-apical approach with resection of the anterior mitral valve leaflet the risk of LVOT obstruction was felt to be low. Preoperative Euroscore II: 6.4, Logistic Euroscore 10.9, STS 7.137.

The procedure was performed via full median sternotomy with routine central bicaval cannulation. A dilated heart with severe left ventricular hypertrophy (LVH) was noted. Custodial cold cardiopledgia was delivered initially via a root vent cannula and subsequently directly to the coronary ostia following a hockey stick aortotomy. There was extensive calcific plaques identified along the back wall of ascending aorta, along the STJ and circumferentially involving the coronary ostia. The aortic valve was trileaflet with extensive leaflet and annular calcification extending onto the aorto-mitral curtain. The aorto-mitral curtain was not amendable to debridement as the calcification extended deep into the tissue. Initial aortic annular measurement permitted a 25 mm valve sizer.

The left atrium (LA) was opened along the intra-atrial groove, however satisfactory visualization of the mitral valve was not possible. We then proceeded to incise the right atrium and inter-atrial septum to improve exposure of the mitral valve (MV) but with limited access to the medial and lateral aspects of the annulus which was then aided by video assisted guidance. Severe bileaflet calcification of the MV involving the annulus in its entirety was noted.

**Fig. 2:**
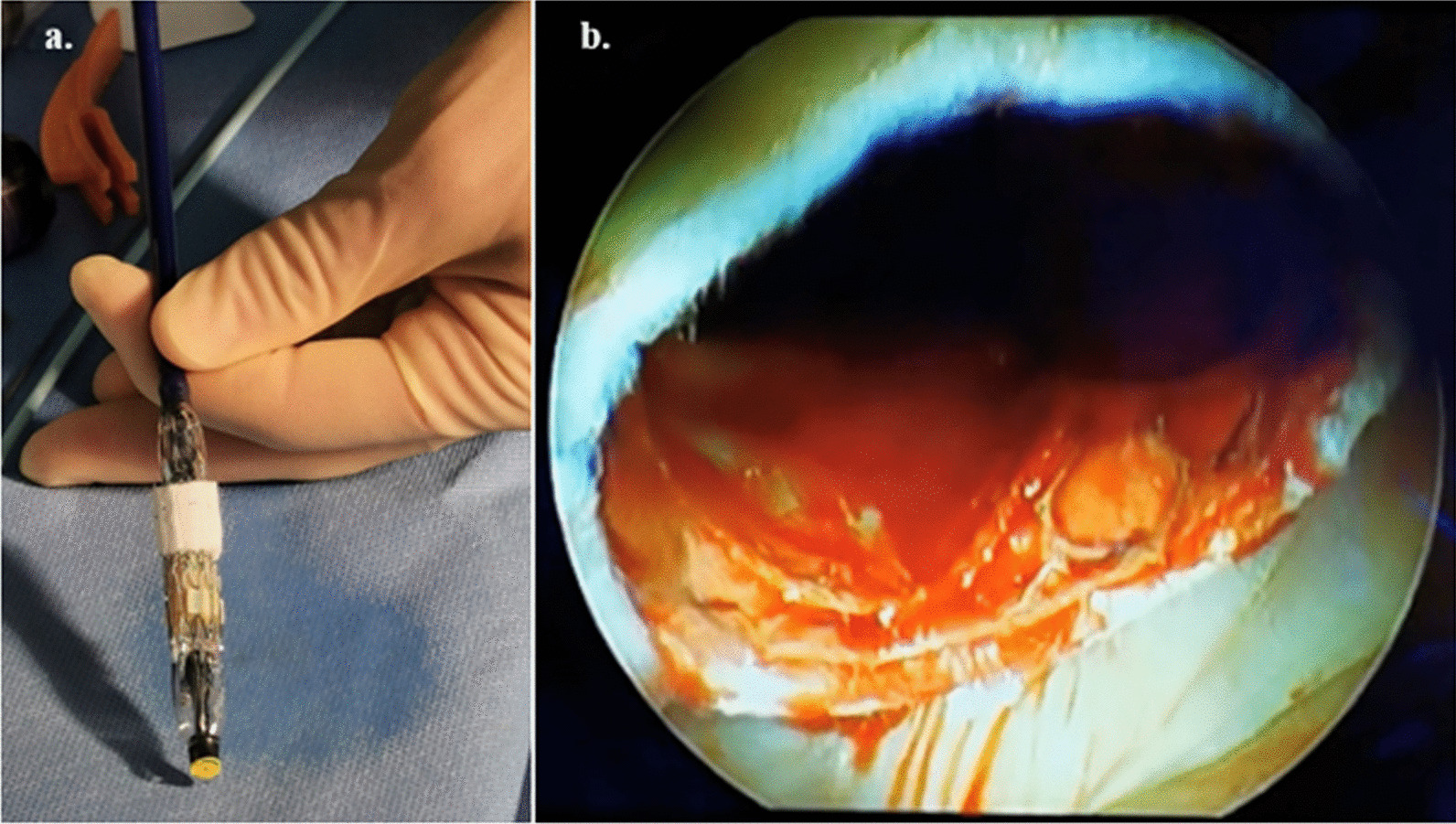
**a** Crimped Sapien 3 Valve augmented with Teflon strip and nose come removed. **b** Intraoperative view-assessment following deployment of the Sapien 3 prosthesis

The anterior MV leaflet and its corresponding subvalvular apparatus was then excised. A size 25 mm Edwards balloon was inflated in the MV orifice with a slightly loose fit noted. We therefore opted to proceed with a size 29 Sapien 3 (Edwards Lifesciensces, Irvine, CA) transcatheter prosthesis. To prevent any paravalvular leak, we sutured a thin Teflon strip onto the skirt of the Sapien 3 valve using 4.0 Prolene (continuous horizontal mattress). The valve was then crimped and deployed (nominal + 2 cc balloon inflation) with both direct vision and video assisted guidance with the orientation slightly angled towards the diaphragm to prevent left ventricular outflow tract (LVOT) obstruction and with the skirt of the valve/Teflon at the level of the native annulus. Figure 2a and b demonstrate the augmented prosthesis crimped and deployed respectively. Due to the limited LA access, we could only secure the valve in 3 positions, (3, 6 and 9 o’clock) rather than run a continuous 4.0 Prolene between the wall of the left atrium and skirt of the prosthesis to prevent valve migration and paravalvular leak (PVL), as previously published [1].

The aortic valve annulus was then resized and continued to accommodate a size 25 mm sizer. A 25 mm Intuity Elite valve was deployed using 3 guiding sutures (inserted higher to prevent interaction between the 2 prosthesis) and balloon inflation with 5 ATM for 10 s. Due to the increased height of the coronary ostia from the annulus this comfortably permitted the prosthesis in a more supra annular position without any encroachment or obstruction. The guiding sutures were tied and a single horizontal mattress suture was inserted along the non-coronary cusp as there was concern about a potential paravalvular leak in this region. Visually there was a clear separation of the two prosthesis with no obstruction or crowding of the LVOT.

The patient was weaned from cardiopulmonary bypass (CPB) on first attempt with minimal inotropic support and intrinsic PPM. Cross-clamp time and CPB time were 214 min and 248 min respectively. The procedural length was protracted by the challenging exposure requiring alteration to the standard approach with video assisted guidance to help deploy the transcatheter valve safely and precisely.

TOE demonstrated good deairing, no PVL along aortic prosthesis, trace of PVL along the A2 segment of the mitral prosthesis, good functioning mitral prosthesis (mean gradient 2 mmHg), good functioning aortic prosthesis (mean gradient 9 mmHg). No LVOT obstruction was observed (mean gradient 4 mmHg) with improved stroke volume from 60 to 100 mls.

**Fig. 3:**
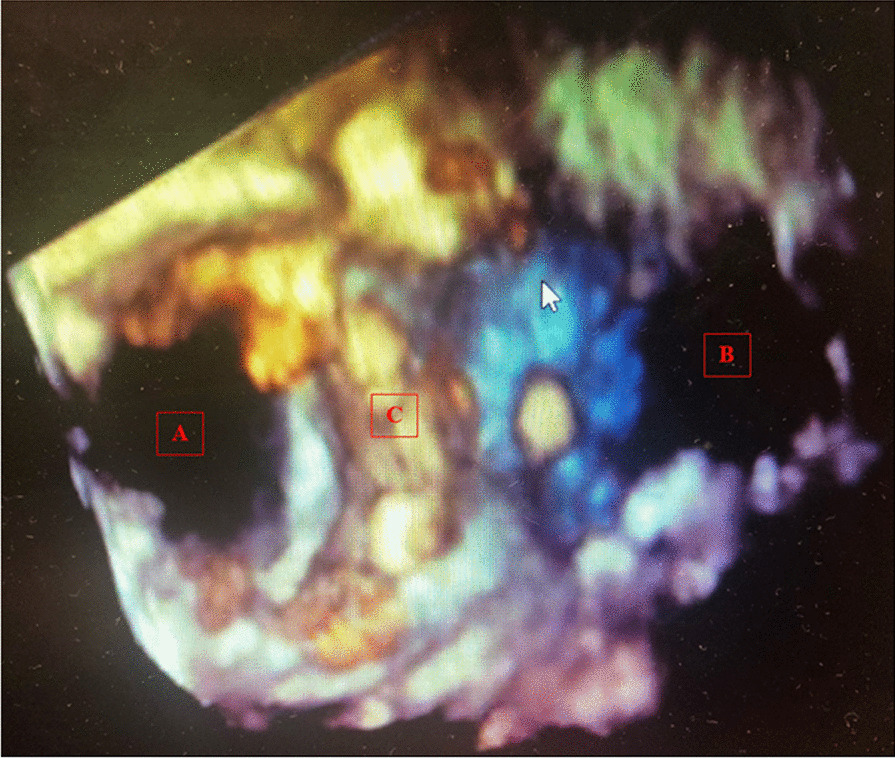
3D transeophageal echocardiogram image demonstrating both Sapien 3 and Intuity elite orifice from within the left ventricle with no impingement, (A) and (B) respectively. (C) Demonstrates the frame of the Sapien 3 prosthesis

Formal transthoracic echocardiogram on the 5th postoperative day demonstrated preserved LV systolic function, mean gradient LVOT 2 mmHg, peak velocity 1 m/s. Mean gradient across prosthetic AV 6 mmHg, peak velocity 1.6 m/s. Mean gradient across prosthetic MV 4 mmHg, peak velocity 2.1 m/s. Figure 3 demonstrates the in-situ relationship of the two prosthesis on 3D ECHO reconstruction in a subgastric view visualizing the valves as they appear from the left ventricle. The patient was discharged from ICU on the second post-operative day and subsequently made an uneventful recovery and is due to be reviewed at the out-patient clinic.

## Discussion and conclusions

This case represents more than mere proof of feasibility. There is an established body of literature supporting the use of each prosthesis with adoption of TMVI in specialist centers now increasing. Similarly, rapid deployment aortic valve technology initially adopted to shorten ischaemia time on CPB has growing evidence of superior haemodynamic performance due to the subannular frame configuring the LVOT to a more circular shape, therefore reducing turbulence and subsequently generating lower transvalvular gradient [2, 3].

As we collectively strive to innovate and advance the field of cardiac surgery, it is vitally important we participate in registries to allow dissemination of data especially with off-label use of existing technologies to validate their use and where possible supported by randomized control trials.

## Data Availability

Not applicable.

## Abbreviations

MAC: Mitral Annular Calcification
TMVI: Transcatheter mitral valve implantation
NYHA: New York Heart Association
CCS: Canadian Cardiovascular Society
BMI: Body Mass Index
AVA: Aortic valve area
MR: Mitral regurgitation
TR: Tricuspid regurgitation
LVH: Left ventricular hypertrophy
LA: Left atrium
MV: Mitral valve
LVOT: Left ventricular outflow tract
PVL: Para valvular leak
CPB: Cardio pulmonary bypass
PPM: Permanent pace maker
ECHO: Echocardiogram
ICU: Intensive Care Unit
CT: Computed tomography
